# Optimizing Finite-Blocklength Nested Linear Secrecy Codes: Using the Worst Code to Find the Best Code

**DOI:** 10.3390/e25101456

**Published:** 2023-10-17

**Authors:** Morteza Shoushtari, Willie Harrison

**Affiliations:** Department of Electrical and Computer Engineering, Brigham Young University, Provo, UT 84602, USA; willie.harrison@byu.edu

**Keywords:** wiretap channel, generalized Hamming weights, dimension/length profile, nested linear codes, equivocation, optimal secrecy code, two-edge LDPC codes, dual codes

## Abstract

Nested linear coding is a widely used technique in wireless communication systems for improving both security and reliability. Some parameters, such as the relative generalized Hamming weight and the relative dimension/length profile, can be used to characterize the performance of nested linear codes. In addition, the rank properties of generator and parity-check matrices can also precisely characterize their security performance. Despite this, finding optimal nested linear secrecy codes remains a challenge in the finite-blocklength regime, often requiring brute-force search methods. This paper investigates the properties of nested linear codes, introduces a new representation of the relative generalized Hamming weight, and proposes a novel method for finding the best nested linear secrecy code for the binary erasure wiretap channel by working from the worst nested linear secrecy code in the dual space. We demonstrate that our algorithm significantly outperforms the brute-force technique in terms of speed and efficiency.

## 1. Introduction

**Figure 1:**
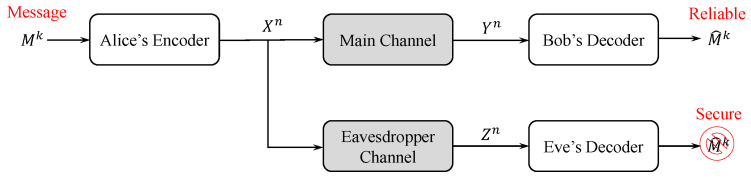
The wiretap channel model.

The wiretap channel introduced by Wyner in [1] and later generalized by Csiszár and Körner in [2] is the most fundamental channel model that has been used to study broadcast security problems in the context of information theory. One version of this channel model is depicted in Figure 1, where the confidential communication occurring over a discrete memoryless main channel is observed by an eavesdropper who has access to a noisy version of the channel input. Later, in [3], Ozarow and Wyner introduced the wiretap channel type II, wherein the eavesdropper is able to select the positions of revealed bits, and they provided a secure coding technique based on coset codes. These channel models have been studied by many authors from the perspectives of security, reliability, and coding construction [4,5,6].

In recent years, coset coding has emerged as an important coding technique in the context of the finite-blocklength regime [7,8,9]. In this regime, the design of the code plays a critical role in achieving high communication rates while balancing the tradeoff between complexity and performance. The effectiveness of coset coding in the finite-blocklength regime has been demonstrated in a wide range of applications, including wiretap channels, broadcast channels, and multiple-access channels [10,11,12,13,14].

The *nested linear code* construction was first presented in [15] to generate a diluted version of the original coset code. Later, in [16,17], the authors proposed a secure error-correcting code based on the nested code construction for the wiretap channel type II, and in [18] the authors considered nested codes based on low-density parity-check (LDPC) codes for the original wiretap channel when both the eavesdropper and main channels are binary erasure channels (BECs).

Generalized Hamming weights (GHW) and the dimension/length profile (DLP), which were first introduced in [19,20], respectively, were two of the first parameters of linear block codes that could be used to characterize the performance of the original linear coset codes, especially over a wiretap channel of type II. Numerous papers have investigated these parameters on various linear codes [21,22,23,24,25,26]. Later, the authors of [16,27] extended these two parameters to nested coding constructions and defined two new formats for them: the relative generalized Hamming weight (RGHW) and relative dimension/length profile (RDLP), which can be used to characterize the security and error-correction performance of nested linear codes. Further studies have shown that with the rank properties of generator and parity-check matrices, the performance of linear codes can also be precisely characterized [28,29,30]. In [29], we utilized rank properties to create and develop a tool for analyzing finite-blocklength wiretap codes based on coset coding over erasure channels, known as an equivocation matrix.

Designing the most secure nested linear codes, referred to as nested linear secrecy codes, to achieve optimal performance in the-finite blocklength regime is still a challenging task, and there is currently no single solution for creating the best codes in this scenario. Identifying these optimal codes would facilitate a comparison of the tradeoffs between complexity and performance for different codes, providing a benchmark for the optimality of other wiretap code designs.

### 1.1. Our Contributions

This paper explores the characteristics of nested linear codes in a wiretap channel model where both the main and eavesdropper channels are BECs. A novel approach is proposed to find the optimal nested linear secrecy codes by using a dual relationship between nested linear codes and their dual codes. Essentially, we demonstrate that instead of searching for the best code directly, it can be found by starting with the worst nested linear secrecy code from the dual space, which is easy to identify. The results demonstrate an efficient and fast technique for finding optimal nested linear secrecy codes.

The main contributions of this work can be summarized as follows:

1. New representation of RGHW: We introduce a new representation of the relative generalized Hamming weight (RGHW) by analyzing the rank properties of parity-check matrices. This innovative approach enables us to accurately predict the security performance of nested linear codes based on rank properties.
2. Equivalence condition evaluation: A comprehensive evaluation of the equivalence codes for nested linear codes is conducted, along with an exploration of its associated properties.
3. Exploration of equivocation curves: We explore and evaluate the equivocation curves of nested linear codes. Notably, we discover that these curves can exhibit both convex and concave characteristics simultaneously, a novel observation in the field. This discovery presents an exciting opportunity to concentrate on codes that effectively balance both secrecy and reliability constraints.
4. Efficient algorithm for best code identification: The main contribution of this paper lies in the development of an algorithm that efficiently identifies the best nested linear secrecy codes. This algorithm surpasses conventional methods in terms of speed and effectiveness.

These contributions not only enrich our understanding of nested linear codes but also enhance their design and deployment for diverse applications.

### 1.2. Organization of the Paper

The rest of the paper is organized as follows. Section 2 consists of preliminary details about the channel model and nested linear coding structure. Section 3 introduces a new expression of the generalized Hamming weight that can be used to quantify the performance of nested linear codes. We also present some properties of nested linear codes in this section. Section 4 describes the behavior of equivocation curves of the nested linear codes. The novel algorithm for finding the best nested linear secrecy codes is explained in Section 5. Section 6 presents a numerical example. In Section 7, we evaluate the complexity of our proposed algorithm. Finally, in Section 8, we present our conclusion.

## 2. Preliminaries

### 2.1. Notation

In this paper, capital letters represent random variables and matrices, lowercase letters represent realizations of these random variables, and calligraphic letters indicate the discrete alphabets associated with the random variables. The distributions p(x) and p(y|x) are probability mass functions. The length of vectors is denoted by superscripts, and sets used as subscripts on matrices specify sub-matrices that include only the columns indexed in the set, i.e., ${\left(H_{1}\right)}_{U}$ is the sub-matrix of $H_{1}$ made up of only the columns with indices in the set *U*. All vectors are row vectors, and all codes are binary. The notation 〚1,γ〛 represents a series of integers ranging from 1 to γ, where γ≥1. The set $R^{n}$ represents all possible revealed-bit patterns over *n* transmitted bits by containing all subsets of 〚1,n〛, whereas the set J∖r indicates the set difference operation and is often read as *J delete r*.

### 2.2. Channel Models

Consider the wiretap channel model in Figure 1. The channels between Alice and Bob and Alice and Eve can be any discrete memoryless channels, but for the purposes of this work, we assume that both channels are BECs, with erasure probabilities of $\epsilon_{m}$ and $\epsilon_{e}$ for the main and eavesdropper channels, respectively. In this model, Alice wants to transmit a secret message $M^{k}$, which is assumed to be chosen uniformly at random from the alphabet $M=F_{2}^{k}$, to Bob through the main channel and wishes to keep it secret as much as possible from a passive eavesdropper (Eve). To achieve this, Alice converts $M^{k}$ into an *n*-bit binary codeword $X^{n}$. The encoding is an invertible one-to-many mapping. This means that no more than one message can be mapped into the same codeword, but each message can be encoded to one of several possible codewords. Bob and Eve observe a noisy version of the transmitted codeword $X^{n}$ through each of their channels, which are denoted by $Y^{n}$ and $Z^{n}$, respectively. Thus, $Y^{n}=Z^{n}={\left\{0,1,?\right\}}^{n}$.

There are two main constraints when utilizing coding over this type of wiretap channel.

1. Reliability constraint for Bob: $\mathrm{Pr}\left(M\neq \hat{M}\right)<\delta_{r}$;
2. Security constraint for Eve: $I\left(M;Z^{n}\right)<\delta_{s}$.

Here, $\delta_{r}$ and $\delta_{s}$ are the desired secrecy and reliability levels, respectively, which can be defined by the system designer. Concisely, the encoding function that maps secret message $M^{k}$ to codeword $X^{n}$ should be such that Bob can decode $M^{k}$ from $Y^{n}$ reliably, and at the same time, Eve receives as little information as possible about $M^{k}$ from $Z^{n}$. The level of secrecy achieved by a code can be quantified by either the average equivocation

(1) $$H\left(M^{k}|Z^{n}\right)=\sum_{z\in Z}p\left(z^{n}\right)H\left(M^{k}|Z^{n}=z^{n}\right),$$

or the average leakage

(2) $$I\left(M^{k};Z^{n}\right)=H\left(M^{k}\right)-H\left(M^{k}|Z^{n}\right).$$

Both of these information-theoretic functions are used to evaluate the performance of a wiretap code in terms of its secrecy. As a result, in this scenario, it is preferable to minimize the average leakage $I(M^{k};Z^{n})$ or maximize the average equivocation $H(M^{k}|Z^{n})$, while also enhancing Bob’s error-correction capabilities, which can be achieved by reducing $H(M^{k}|Y^{n})$.

### 2.3. Nested Linear Codes

The fundamental concept behind the nested linear coding approach is to partition the main code into sub-codes and employ n−k overhead bits to aid in secrecy or reliability as desired. The information rate between Alice and Bob is R=k/n. Let the number of overhead bits assigned to reliability and secrecy be α and *l*, respectively, and let

(3) n=k+α+l.

Let $C_{0}$ be an (n,k+l) linear block code and $C_{1}$ be an (n,l) linear block code. Then, the nested linear code ($C_{0}$, $C_{1}$) is defined, where $C_{0}$ is a *fine code* with rate $R_{0}$ and $C_{1}$ is a *coarse code* with rate $R_{1}$, where $R_{1}\leq R_{0}$, satisfying

(4) $$C_{1}\subseteq C_{0},$$

which means that each codeword of $C_{1}$ is also a codeword of $C_{0}$. Let the l×n matrix $G_{1}$ be the generator matrix, and the (n−l)×n matrix $H_{1}$ be the parity-check matrix for $C_{1}$. The generator matrix $G_{0}$ is defined as follows:

(5) $$G_{0}=\left[\begin{array}{c} G^{\prime }\\ G_{1} \end{array}\right],$$

where $G^{\prime }$ is comprised of *k* linearly independent rows from $F_{2}^{n}$ that are not in $C_{1}$ and make $G_{0}$ a full-rank matrix. The parity-check matrix $H_{1}$ also consists of two sub-matrices such that

(6) $$H_{1}=\left[\begin{array}{c} H^{\prime }\\ H_{0} \end{array}\right],$$

where $H_{0}$ is α×n and forms a basis for the dual space of the rowspace of $G_{0}$. The dimension of the sub-matrix $H^{\prime }$ is k×n. It is important to note that according to the algebraic properties of nested linear codes, $G_{0}{\left(H_{0}\right)}^{T}=0$ and $G_{1}{\left(H_{1}\right)}^{T}=0$.

The encoding process begins by selecting an auxiliary message $m^{\prime }$ uniformly at random from $F_{2}^{l}$ and then computing

(7) $$\begin{array}{cc} x^{n} & =\left[\begin{array}{cc} m & m^{\prime } \end{array}\right]G_{0}=\left[\begin{array}{cc} m & m^{\prime } \end{array}\right]\left[\begin{array}{c} G^{\prime }\\ G_{1} \end{array}\right] \end{array}$$

(8) $$\begin{array}{cc} \; & =mG^{\prime }⊕m^{\prime }G_{1},\; \end{array}$$

where *m* is a *k*-bit secret message. Now, the fine code $C_{0}$ is randomly partitioned into $2^{k}$ disjoint subsets (cosets). The term $mG^{\prime }$ selects the coset, and the term $m^{\prime }G_{1}$ selects the specific codeword from the corresponding coset at random.

Bob uses the following decoding approach to retrieve $M^{k}$ from $Y^{n}$. First, Bob recovers as many erased bits as possible using the parity-check matrix $H^{\prime }$ and obtains an estimated version ${\hat{X}}^{n}$ of $X^{n}$ [31]. Assuming ${\hat{X}}^{n}=X^{n}$, then Bob’s decoder computes the syndrome *S* of ${\hat{X}}^{n}$ as

(9) $$\begin{array}{cc} s & =\hat{x}{\left(H_{0}\right)}^{T}=mG^{\prime }{\left(H_{0}\right)}^{T}⊕m^{\prime }G_{1}{\left(H_{0}\right)}^{T} \end{array}$$

(10) $$\begin{array}{cc} \; & =mG^{\prime }{\left(H_{0}\right)}^{T}.\; \end{array}$$

It is possible to choose matrices such that $G^{\prime }{\left(H_{0}\right)}^{T}$ is the k×k identity; therefore, s=m [32].

To achieve reliability and/or security, both codes $C_{0}$ and $C_{1}$ need to meet specific requirements. In this case, the fine code is primarily responsible for ensuring reliability, while the coarse code is utilized for security purposes. The following section will explore different properties of nested linear codes and examine several parameters that measure the performance of such codes.

## 3. Performance Parameters

This section explores practical metrics to measure the performance of nested linear codes and examines their properties. Consider the (n,n−l) and (n,n−k−l) dual codes of $C_{1}$ and $C_{0}$ and call them $C_{1}^{⊥}$ and $C_{0}^{⊥}$, respectively. $C_{1}^{⊥}$ uses $H_{1}$ as the generator matrix and $G_{1}$ as the parity-check matrix. Hence, the nested linear code $(C_{1}^{⊥}$, $C_{0}^{⊥})$ is the dual code of $(C_{0}$, $C_{1})$. In the dual space, $C_{1}^{⊥}$ serves as the fine code, and $C_{0}^{⊥}$ is the coarse code. The information rate of the nested linear code in both spaces will not change and remains k/n. However, the secrecy and reliability overhead bits will change in the different spaces. In the dual space, α and *l* represent the number of security and reliability bits, respectively [30].

### 3.1. RGHW and RDLP

As previously stated, RGHW and RDLP are extended versions of the GHW and DLP, which can be utilized to characterize the security performance of nested linear codes over the wiretap channel of type II. Let *J* be a subset of 〚1,n〛. A new representation for the RGHW of the nested linear codes can be given as follows.

**Proposition** **1.** *The τth relative generalized Hamming weight of the nested code $\left(C_{0},C_{1}\right)$ can be written as*

(11) $$\begin{array}{cc} \; & M_{\tau }=\min_{1\leq \tau \leq R_{0}-R_{1}}\{|J|:\mathrm{rank}\left({\left(H_{1}\right)}_{J}\right)-\mathrm{rank}\left({\left(H_{0}\right)}_{J}\right)\geq \tau \} \end{array}$$

(12) $$\begin{array}{cc} \; & =n-\max\{|r\left(z^{n}\right)|:\log_{2}N_{0}\left[r\left(z^{n}\right)\right]-\log_{2}N_{1}\left[r\left(z^{n}\right)\right]\geq \tau \}, \end{array}$$

*where $r(z^{n})$ is a revealed-bit pattern over the erasure channel and $N_{0}\left[r\left(z^{n}\right)\right]$ and $N_{1}\left[r\left(z^{n}\right)\right]$ are the number of codewords in $C_{0}$ and $C_{1}$, respectively, that have zeros for all bit locations in the indexed set $r(z^{n})$.*

**Proof.**  In [33], we showed that

(13) $$H\left(M|Z^{n}=z^{n}\right)=\log_{2}N_{0}\left[r\left(z^{n}\right)\right]-\log_{2}N_{1}\left[r\left(z^{n}\right)\right],$$

Since $\left|r(z^{n})\right|$ represents the maximum number of bits that can be revealed while still maintaining at least τ bits of equivocation, the total number of bits minus the maximum revealed bits must equal the minimum number of bits that must be leaked to reveal at least τ bits of information, and the expression (12) is valid. Thus, Equations (11) and (12) represent two equivalent expressions for the τth relative generalized Hamming weight of the nested linear code. □

### 3.2. Rank Properties and the Equivocation Matrix

Let $r\left(z^{n}\right)=\left\{i:z_{i}\neq \;?\right\}$, where $z_{i}$ is the observation of the *i*th bit of the codeword *x* over the eavesdropper’s BEC and “?” denotes an erased bit. Also, let I=〚1,n〛. According to the results of [30,33], we showed that the exact equivocation for the observation $z^{n}$ over a binary erasure channel (BEC), given the coding scheme presented in Section 2.3, is

(14) $$\begin{array}{cc} H(M|Z^{n}=z^{n}) & =k-\mathrm{rank}\;\left[{\left(G_{0}\right)}_{r(z^{n})}\right]+\mathrm{rank}\;\left[{\left(G_{1}\right)}_{r(z^{n})}\right] \end{array}$$

(15) $$\begin{array}{cc} \; & =\mathrm{rank}\;\left[{\left(H_{1}\right)}_{I\setminus r(z^{n})}\right]-\mathrm{rank}\;\left[{\left(H_{0}\right)}_{I\setminus r(z^{n})}\right]. \end{array}$$

Thus, in terms of code design for security and reliability, a revealed-bit pattern $r(z^{n})$ is secure if and only if $\mathrm{rank}\left({\left(G_{0}\right)}_{r(z^{n})}\right)=\mathrm{rank}\left({\left(G_{1}\right)}_{r(z^{n})}\right)$. Furthermore, for reliability, the message information is obtained if and only if $\mathrm{rank}\left({\left(G_{0}\right)}_{r(z^{n})}\right)-\mathrm{rank}\left({\left(G_{1}\right)}_{r(z^{n})}\right)=k$. The following definition is from [30].

**Definition** **1.** *The (k+1)×(n+1) equivocation matrix A for the linear block code C is a matrix where each entry ($a_{e,\mu }$) counts the number of revealed-bit patterns of size μ that maintain e bits of equivocation.*

There are nμ different patterns that can be used to reveal μ bits of *n* transmitted codeword bits over the erasure channel, and the bottom left entry of *A* is $a_{0,0}$.

### 3.3. Equivalence of Nested Linear Codes

**Lemma** **1.** Let $\left(C_{0}^{*},C_{1}^{*}\right)$ and $\left(C_{0}^{⊛},C_{1}^{⊛}\right)$ be two nested linear codes with generator matrices $G_{0}^{*}$ and $G_{0}^{⊛}$, respectively. These two nested linear codes are equivalent if there exist two invertible scrambling matrices $F_{1}$ and $F_{2}$ and permutation matrix P, such that

(16) $$G_{0}^{⊛}=\left[\begin{array}{cc} F_{1} & \underline{0}\\ \underline{0} & F_{2} \end{array}\right]\times G_{0}^{*}\times P,$$

*where $F_{1}$ and $F_{2}$ are k×k and l×l full-rank matrices, respectively, and $\underline{0}$ is a zero matrix.*

Note that, in general, codes are equivalent if the sets of codewords are the same up to the permutation of bit order in the codewords.

**Proof.**  We know that the space spanned by the rows of $G_{1}^{*}$ is the same as the space spanned by the rows of $F_{2}G_{1}^{*}$ (and similarly for the space spanned by the rows of ${G^{\prime }}^{*}$ and $F_{1}{G^{\prime }}^{*}$). The multiplication by *P* changes only the order of bits in codewords and the mapping of specific messages to specific codewords but achieves equivalence. □

**Lemma** **2.** If generator matrices $G_{0}^{*}$ and $G_{0}^{⊛}$ correspond to respective equivalent nested codes $\left(C_{0}^{*},C_{1}^{*}\right)$ and $\left(C_{0}^{⊛},C_{1}^{⊛}\right)$, then the RGHW, RDLP, and equivocation matrices for the two codes are identical.

**Proof.**  According to Lemma 1, two nested linear codes $\left(C_{0}^{*},C_{1}^{*}\right)$ and $\left(C_{0}^{⊛},C_{1}^{⊛}\right)$ are equivalent if $G_{0}^{*}$ can be converted into $G_{0}^{⊛}$ using simple linear operations over rows and/or column pivots. These basic operations produce the same set of codewords from the new generator matrices up to a consistent bit permutation in the codeword sets. Thus, (11) and (12) are the same for both codes for all τ, and the equivalence for the RDLP is similarly trivial. For the equivalence of equivocation matrices, every $r(z^{n})$ for code $\left(C_{0}^{*},C_{1}^{*}\right)$ maps to a unique revealed-bit pattern for $\left(C_{0}^{⊛},C_{1}^{⊛}\right)$ of the same size such that (14) is equivalent. □

Previous research, including [16,17], has analyzed the bounds on the RGHW and RDLP of nested linear codes $\left(C_{0},C_{1}\right)$ to aid in constructing nested linear secrecy codes. Furthermore, studies such as [29,30] enable comparisons between the performance of nested linear codes on specific sizes. Even with these results, the challenge of finding optimal nested linear secrecy codes remains unsolved and requires a brute-force search.

## 4. Concavity and Convexity of Equivocation Curves

The equivocation quantifies Bob and Eves’ uncertainty about the secret message $M^{k}$ after observing $Y^{n}$ and $Z^{n}$, respectively. We may want to maximize the equivocation for security constraints or minimize it for reliability limitations, depending on the system requirements. In the noiseless main channel model where reliability constraints are not considered and all overhead bits are allocated for security purposes, the equivocation of the nested linear code $\left(C_{0},C_{1}\right)$ is always a concave function of ϵ [32]. However, when both reliability and security are important, such as in the case of a noisy main channel, our simulation results indicate a different behavior compared to the noiseless main channel case.

**Lemma** **3.** *Consider the nested linear code $\left(C_{0},C_{1}\right)$ of rate $R_{0}$ and $R_{1}$, respectively. Assume that this pair of linear codes is used to transmit a k-bit message m over the binary erasure wiretap channel. The equivocation curve that can be achieved by nested coding construction can be concave, convex, or both as a function of ϵ.*

The proof follows directly from Theorem 2.7.4 [34] and is included here for completeness.

**Proof.**  H(M) is a concave function of p(m), and

(17) $$\begin{array}{cc} I\left(M^{k};Z^{n}\right)=I\left(Z^{n};M^{k}\right) & =H\left(Z^{n}\right)-H\left(Z^{n}|M^{k}\right) \end{array}$$

(18) $$\begin{array}{cc} \; & =H\left(Z^{n}\right)-\sum_{m}p\left(m\right)H\left(Z^{n}|M^{k}=m\right). \end{array}$$

If p(z|m) is fixed, then p(z) is a linear function of p(m); hence, $H(Z^{n})$ is also a concave function of p(m), and the second term of (17) is a linear function of p(m). The difference is then a concave function of p(m). Moreover, the conditional entropy $H(Z^{n}|M^{k})$ of p(z|m) for a fixed p(m) will be concave, and the difference of two concave functions can either be concave, convex, or both. □

Simulation results show that there are indeed three distinct equivocation curve behaviors for nested linear codes $\left(C_{0},C_{1}\right)$, as follows:

- Convex equivocation curve: These codes are appropriate for situations when $\delta_{r}$ is small; thus, Alice may purposefully use a nested linear code of this nature to improve Bob’s ability to correct errors.
- Concave equivocation curve: If $\delta_{s}$ is small, these codes give Alice the ability to keep data as secure as possible from the eavesdropper.
- Convex/concave equivocation curve: The more desirable and interesting codes are those that provide both reliability for Bob and confusion for Eve, in scenarios where Bob and Eve experience erasure with different rates. These codes can effectively balance both constraints as required, resulting in a convex/concave equivocation curve.

Simulations of (1) were completed using (14) and considering all possible erasure patterns $r(z^{n})$. Curves were plotted as a function of the erasure probability ϵ, noting that $p\left(z^{n}\right)=\epsilon^{n-|r(z^{n})|}{\left(1-\epsilon \right)}^{\left|r(z^{n})\right|}$. This examination of the behavior of equivocation curves enhanced our comprehension of nested linear codes, unveiling the dual relationship between error correction capabilities and security attributes. Furthermore, our simulations demonstrated that the number of overhead bits allocated to security or reliability can have a significant impact on the shape and number of the equivocation curves. In particular, increasing the number of overhead bits allocated to security (*l*) can lead to an increase in the number of concave curves. This observation is consistent with the fact that adding more security overhead bits to the code will result in a higher level of confusion for the eavesdropper. Similarly, increasing the number of overhead bits allocated to reliability (α) can lead to an increase in the number of convex curves and affect their shape, as more reliability overhead bits will provide Bob with better error correction capabilities. Overall, our results highlight the importance of carefully balancing the allocation of overhead bits between security and reliability to achieve the desired level of secrecy and reliability for the system. Additionally, we showed that there exist codes that can balance both restrictions effectively (codes with convex/concave equivocation curves).

**Figure 2:**
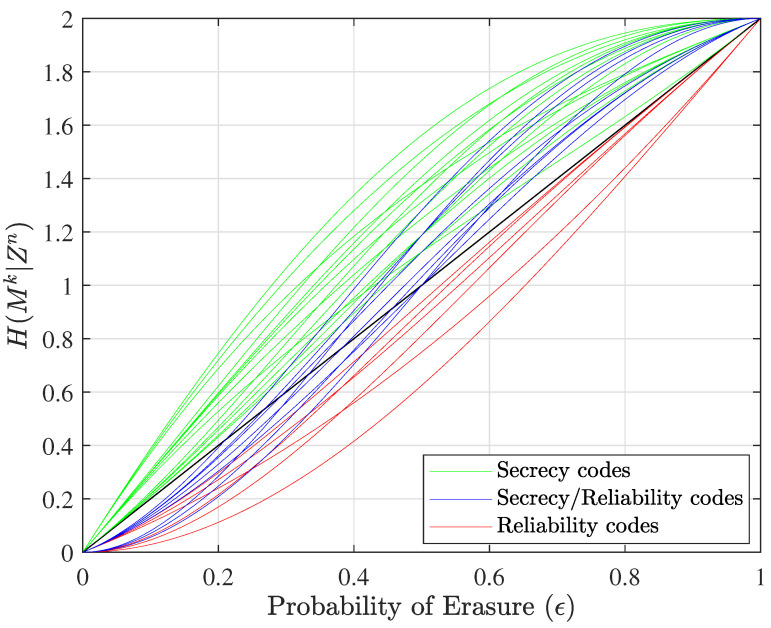
Equivocation curves of all nested linear codes versus ϵ (the nested linear codes designed for n=5, k=2, l=2, and α=1). The green pair of codes are good for security purposes, and the red pair of codes are suitable for reliability.

**Figure 3:**
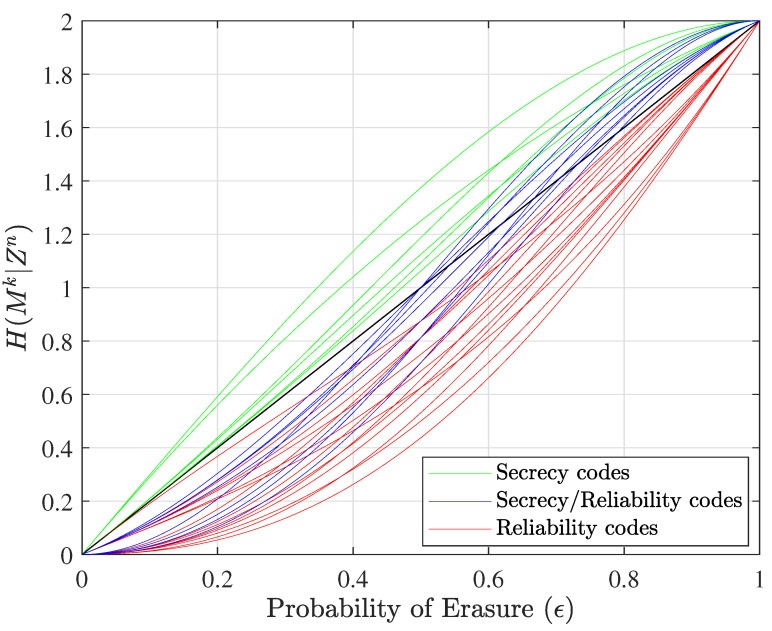
Equivocation curves of all nested linear codes when n=5, k=2, l=1, and α=2 (referring to the dual nested linear codes).

These types of codes are represented, respectively, with red, green, and blue equivocation curves in Figure 2 for the n=5, k=2, l=2, and α=1 case, and in Figure 3 for the corresponding dual case, where *l* and α change their responsibilities, which means that the number of overhead bits allocated to security will be α=2, and the number of overhead bits allocated to reliability will be l=1. This change in the allocation of overhead bits results in a different set of equivocation curves. The probability of erasure, ϵ, refers to both $\epsilon_{m}$ and $\epsilon_{e}$ to show performance for all users on the same plot.

## 5. Finding the Best Nested Linear Secrecy Codes

In this section, we propose a coding construction algorithm to generate the best nested linear secrecy code according to the equivocation by taking advantage of the dual relationship between nested linear codes. In essence, we show that the difficult search for the best code can be computed instead by the easy search for the worst nested linear secrecy code in the dual space. The concept of the *worst* and *best* refers to nested linear codes with the lowest and highest security level, respectively, among all possible nested linear codes for a particular size. The general algorithm is provided here, and an example is given in Section 6.

**Algorithm** **1.** *This algorithm demonstrates how to construct the best nested linear secrecy codes $\left(C_{0},C_{1}\right)$ through the construction of the worst nested linear secrecy codes $\left(C_{1}^{⊥},C_{0}^{⊥}\right)$ using the subsequent steps:*

- *The first phase:*
  - *Generate the worst secrecy code $\left(C_{0}^{⊥}\left(n,\alpha \right)\right)$ with generator matrix $H_{0}$. The general schematic of the worst $H_{0}$ can be found in (30) in Section 6.*
  - *Generate $H^{\prime }$ by searching k random vectors from $F_{2}^{n}$ with the following considerations:*
    ∗ *For most patterns of revealed bits $r(z^{n})$, the rank of ${\left(H_{1}\right)}_{r(z^{n})}$ should be as large as possible compared to the rank of ${\left(H_{0}\right)}_{r(z^{n})}$.*
    ∗ *$H_{1}$ should remain a full-rank matrix.*
- *The second phase:*
  - *The best generator matrix $G_{1}$ for security code $C_{1}\left(n,l\right)$ is equal to the basis of the dual space of the rowspace of $H_{1}$.*
  - *Choose k rows from a basis of the dual space of $H_{0}$ as $G^{\prime }$, with a consideration of the following:*
    ∗ *For most patterns of $r(z^{n})$, the rank of ${\left(G_{0}\right)}_{r(z^{n})}$ should be equal to the rank of ${\left(G_{1}\right)}_{r(z^{n})}$.*
    ∗ *$G_{0}$ remains a full-rank matrix.*

**Proof.**  According to our result in [30], it can be deduced that minimizing the equivocation in the dual space of the nested linear codes leads to the maximization of equivocation in the original space of the nested linear code.

In particular, we have:

(19) $$\begin{array}{cc} H(M) & =\underset{I(M^{k};Z^{n}=r\left(z^{n}\right))}{\underset{︸}{\mathrm{rank}\;\left[{\left(G_{0}\right)}_{r(z^{n})}\right]+\mathrm{rank}\;\left[{\left(G_{1}\right)}_{r(z^{n})}\right]}}+ \end{array}$$

(20) $$\begin{array}{cc} \; & \underset{H(M^{k}|Z^{n}=r\left(z^{n}\right))}{\underset{︸}{\mathrm{rank}\;\left[{\left(H_{1}\right)}_{I\setminus r(z^{n})}\right]+\mathrm{rank}\;\left[{\left(H_{0}\right)}_{I\setminus r(z^{n})}\right]}}, \end{array}$$

or, equivalently,

(21) $$\begin{array}{cc} H(M) & =\underset{H(M^{k}|Z^{n}=I\setminus r\left(z^{n}\right))}{\underset{︸}{\mathrm{rank}\;\left[{\left(G_{0}\right)}_{r(z^{n})}\right]+\mathrm{rank}\;\left[{\left(G_{1}\right)}_{r(z^{n})}\right]}}+ \end{array}$$

(22) $$\begin{array}{cc} \; & \underset{I(M^{k};Z^{n}=I\setminus r\left(z^{n}\right))}{\underset{︸}{\mathrm{rank}\;\left[{\left(H_{1}\right)}_{I\setminus r(z^{n})}\right]+\mathrm{rank}\;\left[{\left(H_{0}\right)}_{I\setminus r(z^{n})}\right]}}. \end{array}$$

Therefore, by constructing the nested linear code that minimizes the equivocation in the dual space, we can generate the best nested linear secrecy code within the code space. □

**Figure 4:**
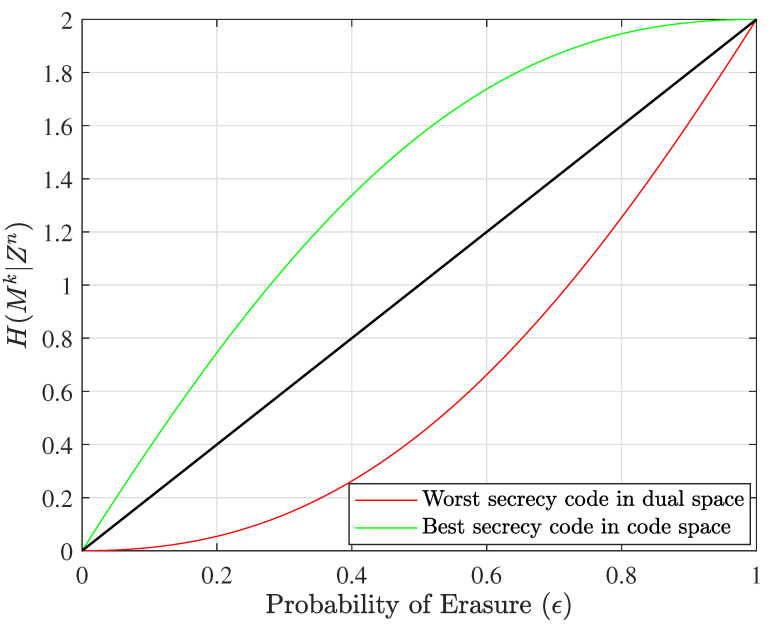
The best nested linear secrecy code when n=5, k=2, l=2, and α=1, along with the worst nested linear code for secrecy in the dual space.

This algorithm can be better understood with reference to an example; hence, the results of this algorithm for a specific size are shown in Figure 4, and the details of this example are given in the next section.

## 6. Numerical Example

Consider the nested code $\left(C_{0},C_{1}\right)$ with the rate $R_{0}=4/5$ and $R_{1}=2/5$, respectively. In other words, n=5, k=2, l=2, and α=1. In this example, the information rate is equal to R=2/5. Let

(23) $$G_{0}=\left[\begin{array}{c} G^{\prime }\\ G_{1} \end{array}\right]=\left[\begin{array}{ccccc} 0 & 1 & 0 & 1 & 0\\ 1 & 0 & 0 & 1 & 1\\ 1 & 0 & 0 & 0 & 1\\ 0 & 1 & 0 & 1 & 1 \end{array}\right],$$

and

(24) $$H_{1}=\left[\begin{array}{c} H^{\prime }\\ H_{0} \end{array}\right]=\left[\begin{array}{ccccc} 0 & 1 & 0 & 1 & 0\\ 1 & 1 & 0 & 0 & 1\\ 0 & 0 & 1 & 0 & 0 \end{array}\right].$$

The RGHW and equivocation matrix for this nested code are equal to

(25) $$\begin{array}{cc} \; & M_{\tau }\left(C_{0},C_{1}\right)=\left\{1,\;2\right\} \end{array}$$

(26) $$\begin{array}{cc} \; & A=\left[\begin{array}{cccccc} 1 & 5 & 9 & 5 & 0 & 0\\ 0 & 0 & 1 & 5 & 4 & 0\\ 0 & 0 & 0 & 0 & 1 & 1 \end{array}\right], \end{array}$$

and for the dual nested code are equal to

(27) $$\begin{array}{cc} \; & M_{\tau }\left(C_{1}^{⊥},C_{0}^{⊥}\right)=\left\{2,\;4\right\} \end{array}$$

(28) $$\begin{array}{cc} \; & A^{⊥}=\left[\begin{array}{cccccc} 1 & 1 & 0 & 0 & 0 & 0\\ 0 & 4 & 5 & 1 & 0 & 0\\ 0 & 0 & 5 & 9 & 5 & 1 \end{array}\right]. \end{array}$$

Figure 2 illustrates the equivocation curves of all unique nested linear codes in this specific size. It should be noted that the number of green equivocation curves is greater than the number of red equivocation curves because in this example we assume that two of the three overhead bits are assigned to security (l=2) and just one bit is assigned to reliability (α=1). If we evaluate nested linear codes in the dual space when l=1 and α=2, we can see that the number of equivocation curves for nested codes that provide an error-correction capability is greater than the number for nested codes that offer a security capability (green equivocation curves). Figure 3 shows the equivocation curves for dual nested linear codes.

We now aim to determine how we can identify the optimal nested linear secrecy code $\left(C_{0},C_{1}\right)$ at this specific size using the algorithm outlined in Algorithm 1. To start, we need to build the generator matrix $H_{0}$ for code $C_{0}^{⊥}$ with the worst rank properties. We know that $H_{0}$ must be a full-rank matrix with the most zero columns, which results in a zero rank in most collections of columns. Hence, $H_{0}$ is

(29) $$H_{0}=\left[1\;0\;0\;0\;0\right].$$

In general,

(30) $$H_{0}=\left[V_{\alpha \times \alpha }{\underline{0}}_{\alpha \times n-\alpha }\right],$$

where *V* and $\underline{0}$ are the identity and zero matrices, respectively. Then, we need to generate $H^{\prime }$ by searching *k* random vectors from $F_{2}^{n}$, with the consideration of the specific criteria as mentioned in Algorithm 1. Let

(31) $$H_{1}=\left[\begin{array}{c} H^{\prime }\\ H_{0} \end{array}\right]=\left[\begin{array}{ccccc} 0 & 1 & 1 & 0 & 1\\ 1 & 1 & 0 & 1 & 0\\ 1 & 0 & 0 & 0 & 0 \end{array}\right].$$

In the second phase, we can generate $G_{1}$ from the dual space of $H_{1}$, which is equal to

(32) $$G_{1}=\left[\begin{array}{ccccc} 0 & 1 & 1 & 1 & 0\\ 0 & 0 & 1 & 0 & 1 \end{array}\right].$$

Now, we need to select *k* rows from the basis of the dual space of $H_{0}$ as follows:

(33) $$\left[\begin{array}{ccccc} 0 & 1 & 0 & 0 & 0\\ 0 & 0 & 1 & 0 & 0\\ 0 & 0 & 0 & 1 & 0\\ 0 & 0 & 0 & 0 & 1 \end{array}\right],$$

Additionally, we must be sure that $G_{0}$ is a full-rank matrix and

(34) $$\mathrm{rank}\left({\left(G_{0}\right)}_{r(z^{n})}\right)=\mathrm{rank}\left({\left(G_{1}\right)}_{r(z^{n})}\right)$$

as much as possible for most patterns of $r(z^{n})$, so

(35) $$G_{0}=\left[\begin{array}{c} G^{\prime }\\ G_{1} \end{array}\right]=\left[\begin{array}{ccccc} 0 & 1 & 0 & 0 & 0\\ 0 & 0 & 1 & 0 & 0\\ 0 & 1 & 1 & 1 & 0\\ 0 & 0 & 1 & 0 & 1 \end{array}\right].$$

The generator matrix $G_{0}$ outperforms other generator matrices in terms of security performance. The equivocation matrix of this example is equal to (26), and Figure 4 depicts the equivocation curves of the worst nested linear secrecy codes in the dual space and the best nested linear secrecy codes in the code space.

In the following section, we will analyze the computational complexity of our proposed algorithm for finding the optimal nested linear secrecy code and compare it with the computational complexity of traditional approaches (the brute-force method).

## 7. Computational Complexity Analysis

The number of distinct generator matrices $G_{0}$ that can be chosen such that the resulting matrix is full-rank can be calculated as

(36) $$\prod_{i=0}^{(n-\alpha )-1}\left(2^{n}-2^{i}\right),$$

where *n* is the number of codeword bits and α is the number of overhead bits allocated to the reliability. This equation gives the total number of different nested linear codes ($C_{0},C_{1}$) for a given size, which for the example explored in Section 6 is 624,960. However, not all of these nested linear codes are unique. Based on Lemma 1, some nested linear codes may be equivalent and have the same performance, while others are unique and cannot be transformed into each other through equivalent operations on generator matrices. Therefore, the total number of unique nested linear codes can be lower than the total number of different nested linear codes. For the example in the previous section, the number of unique generator matrices $G_{0}$ is 256, which is much smaller than the total number of different nested linear codes. However, finding equivalent codes itself is a complex problem, and it is not guaranteed that we can always identify all equivalent codes.

The traditional approach to finding the best nested linear secrecy codes involves a brute-force search over all possible generator matrices $G_{0}$, which are (k+l)×n. This means that for a given size of nested linear code, all possible generator matrices must be formed and their performance calculated. Then, all codes must be compared based on their equivocation to find the best nested linear secrecy code. Using this approach, $2^{(k+l)n}$ generator matrices must be formed. Lemma 1 can be used to identify equivalent generators, but all of them must be examined at some level. In contrast, our proposed approach fixes the matrix $H_{0}$ in the dual space and only requires a search for different patterns of the matrix $H^{\prime }$, which is k×n, with the consideration of the two restrictions explained in Algorithm 1. We can throw out a number of $H^{\prime }$ candidates due to the fixed form of $H_{0}$, e.g., $H^{\prime }$ matrices with any number of zero columns and/or $H^{\prime }$ matrices that do not result in a full-rank $H_{1}$. This significantly reduces the search space and computational complexity compared to the traditional approach. Fewer than $2^{kn}$$H^{\prime }$ matrices must be compared.

In summary, our proposed approach of searching for the worst code instead of the best code was shown to be easier and more efficient, requiring fewer resources. This is because the generator matrix of the worst linear code $C_{0}^{⊥}$ has as many zero columns as possible, making it easier to construct. This improvement in efficiency compared to the full brute-force search method could have important implications for the design of reliable and secure communication systems in practical settings.

**Figure 5:**
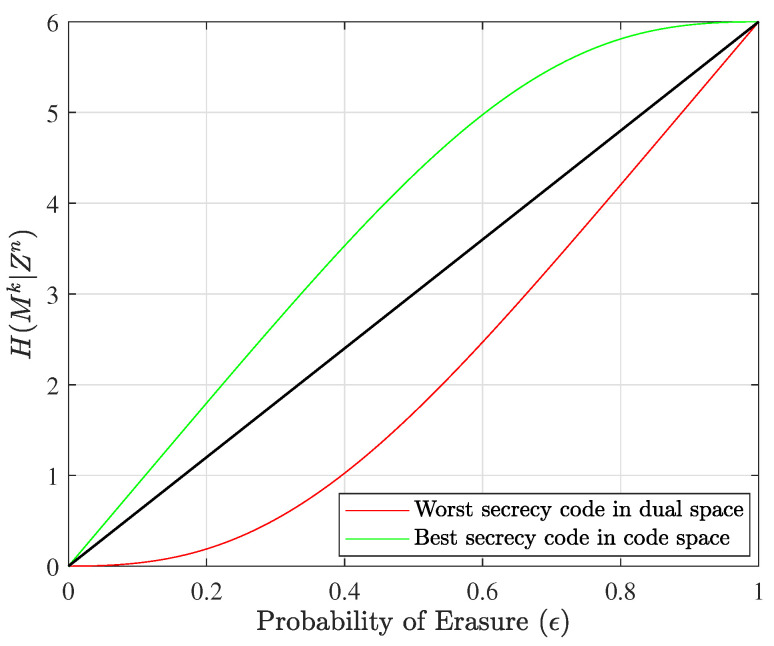
The best nested linear secrecy code when n=12, k=6, l=3, and α=3, along with the worst nested linear secrecy code in the dual space.

**Figure 6:**
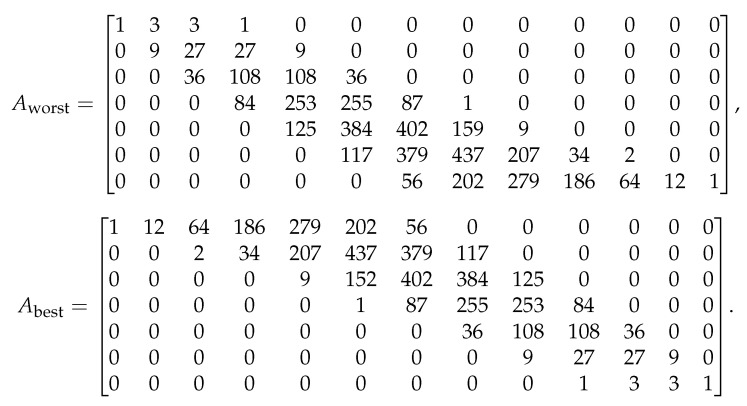
The equivocation matrices $A_{\mathrm{worst}}$ and $A_{\mathrm{best}}$ for the worst and best nested linear secrecy codes, respectively, when n=12, k=6, l=3, and α=3.

On a personal laptop, it is possible to find best codes up to blocklength 12 with little issue, and we show in Figure 5 the results for the best and worst nested linear secrecy codes with n=12, k=6, l=3, and α=3. The best and worst equivocation matrices for this example are given in Figure 6.

**Figure 7:**
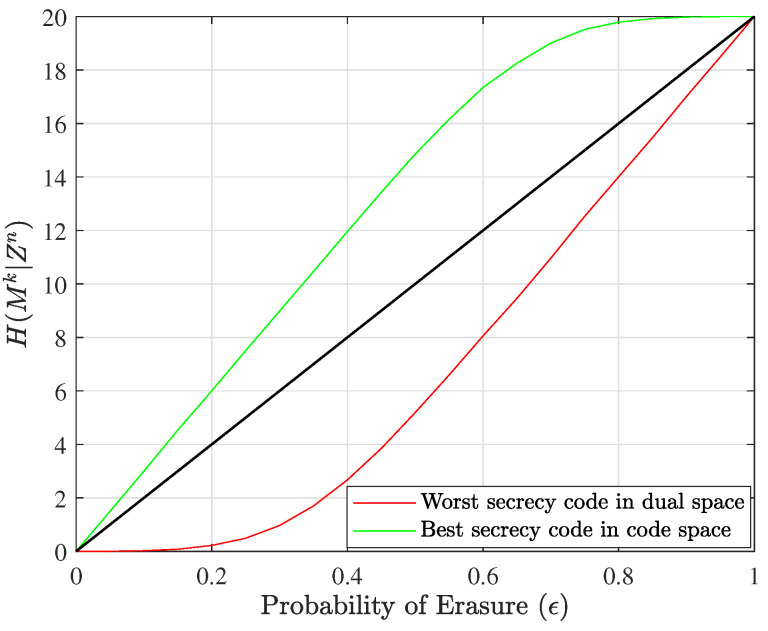
The best nested linear secrecy code for the parameters n=40, k=20, l=10, and α=10, as well as the worst nested linear secrecy code in the dual space.

Note that although there is a marked increase in efficiency for identifying best codes by first finding worst codes in the dual space, Algorithm 1 still requires a brute-force search in choosing the elements of $H^{\prime }$. Thus, for larger code sizes, we still have limitations in finding best codes. In Figure 7, we present performance curves for one set of candidate codes when n=40, k=20, l=10, and α=10. The candidate was found by choosing random columns to fill out $H^{\prime }$ and checking for full rank, as depicted in Algorithm 1. We leave the identification of large optimal codes as an open problem.

## 8. Conclusions and Future Study

In this study, we analyzed the properties of nested linear codes in the presence of a noisy wiretap channel model and derived a new expression for the relative generalized Hamming weight of these codes. We showed that there are three distinct behaviors in terms of equivocation in this coding scheme. Moreover, we proposed a code design algorithm to find the worst nested linear secrecy code, which is constructed by identifying the code with the lowest security in the dual space. Our results demonstrated that this approach is more efficient and quicker in producing optimal nested linear secrecy codes compared to brute-force methods.

Overall, the findings of this paper contribute to the development of reliable and secure communication systems in practical settings. The ability to efficiently design secure nested linear codes can enhance the privacy and security of communication channels, which is of great importance in various applications, such as wireless communication, network security, and cryptography. Future work could explore the applicability of our proposed algorithm to larger blocklengths and investigate its performance in other channel models. 

## Data Availability

Not applicable.

## Abbreviations

The following abbreviations are used in this manuscript:

BEC	Binary erasure channel
LDPC	Low-density parity-check
GHW	Generalized Hamming weights
DLP	Dimension/length profile
RGHW	Relative generalized Hamming weight
RDLP	Relative dimension/length profile
